# *in vivo* localization of the neuronal ceroid lipofuscinosis proteins, *CLN3* and *CLN7*, at endogenous expression levels

**DOI:** 10.1016/j.nbd.2017.03.015

**Published:** 2017-07

**Authors:** Alamin Mohammed, Megan B. O'Hare, Alice Warley, Guy Tear, Richard I. Tuxworth

**Affiliations:** a Institute of Cancer and Genomic Sciences, College of Medical and Dental Sciences, University of Birmingham, Birmingham B15 2TT, UK; b Department of Developmental Neurobiology, King's College London, London SE1 1UL, UK; c Centre for Ultrastructural Imaging, King's College London, London, SE1 1UL, UK

**Keywords:** *CLN3*, *CLN7*, MFSD8, Localization, Neuronal ceroid lipofuscinosis, Batten disease, *Drosophila*

## Abstract

The neuronal ceroid lipofuscinoses are a group of recessively inherited, childhood-onset neurodegenerative conditions. Several forms are caused by mutations in genes encoding putative lysosomal membrane proteins. Studies of the cell biology underpinning these disorders are hampered by the poor antigenicity of the membrane proteins, which makes visualization of the endogenous proteins difficult. We have used *Drosophila* to generate knock-in YFP-fusions for two of the NCL membrane proteins: *CLN7* and *CLN3*. The YFP-fusions are expressed at endogenous levels and the proteins can be visualized live without the need for overexpression. Unexpectedly, both *CLN7* and *CLN3* have restricted expression in the CNS of *Drosophila* larva and are predominantly expressed in the glia that form the insect blood-brain-barrier. *CLN7* is also expressed in neurons in the developing visual system. Analogous with murine *CLN3*, *Drosophila CLN3* is strongly expressed in the excretory and osmoregulatory Malpighian tubules, but the knock-in also reveals unexpected localization of the protein to the apical domain adjacent to the lumen. In addition, some *CLN3* protein in the tubules is localized within mitochondria. Our *in vivo* imaging of *CLN7* and *CLN3* suggests new possibilities for function and promotes new ideas about the cell biology of the NCLs.

## Highlights

• YFP-*CLN3* and YFP-*CLN7* knock-in fusions generated in *Drosophila*
• *CLN7* and *CLN3* both expressed in glia but have limited neural expression
• *CLN7* recruited to post-synaptic density of neuromuscular junctions
• *CLN7* and *CLN3* both expressed in Malpighian tubules
• *CLN3* localizes to the apical domain of tubules and to mitochondria

## Graphical abstract

**Figure:**
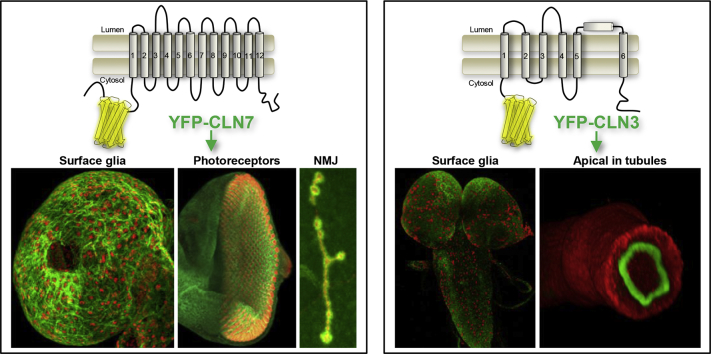

## 1 Introduction

The neuronal ceroid lipofuscinoses (NCLs) are a collection of inherited neurodegenerative lysosomal storage disorders predominantly affecting children (Jalanko and Braulke, 2009). They share symptoms that include visual failure, seizures, psychiatric and behavioral changes and a progressive decline in mental and motor functions followed by premature death. The NCLs have a common histopathological hallmark: accumulation of autofluorescent lysosomal storage material in most cells, including neurons, indicative of lysosomal dysfunction or failure. Another common histopathological feature of the disorders is an early glial activation, which precedes selective neuronal loss (reviewed in Cooper et al., 2015).

The NCLs are recessively inherited monogenic disorders (with the exception of one rare adult-onset autosomal dominant form). To date mutations have been identified in 14 genes responsible for NCL with varying ages of onset. These encode soluble intra-lysosomal proteins and enzymes (*CLN1*, *CLN2*, *CLN5*, *CLN10* and *CLN13*), late endosomal/lysosomal transmembrane proteins (*CLN3*, *CLN7* and *CLN1*2), ER/ERGIC membrane proteins (*CLN6* and *CLN8*), cytosolic proteins (*CLN4* and *CLN14*) and an extracellular protein (*CLN11*) (reviewed in Carcel-Trullols et al., 2015). The cell biology underpinning the NCLs is not well understood despite many years of study, nor is it clear why pathology is almost entirely restricted to the CNS despite many of the *CLN* genes being widely expressed. We have turned to the fruit fly, *Drosophila*, to study two of the *CLN* genes encoding putative lysosomal membrane transporters, *CLN7* and *CLN3*. *Drosophila* expresses only a subset of the *CLN* genes and we hypothesized that these are likely to have core functions conserved in vertebrates.

Mutations in *CLN7/MFS-domain containing 8* (*CLN7/MFSD8*) are responsible for late-infantile onset NCL (or CLN7 disease), with disease onset at 1.5–5 years of age (Kousi et al., 2009). The CLN7 protein is predicted to be a member of the multi-facilitator superfamily of transporters, each of which has twelve membrane spanning domains (Siintola et al., 2007). However, its function and any possible substrate it may transport remains unknown. In cell culture experiments with tagged forms, CLN7 protein is localized primarily in lysosomes (Sharifi et al., 2010, Siintola et al., 2007, Steenhuis et al., 2010) and has been identified by lysosomal proteomics (Chapel et al., 2013). Consistent with this, phenotypes in mutant mice suggest a role for CLN7 in autophagy (Brandenstein et al., 2016). In a related project that will be reported elsewhere, we have identified neurodevelopmental defects in *CLN7* mutant *Drosophila* (O'Hare, Mohammed, Tuxworth and Tear, in prep).

Mutations in the *CLN3* gene lead to Juvenile NCL, the most common form of NCL (also known as Batten disease or CLN3 disease) with onset usually at 5–7 years of age (Lerner et al., 1995). The *CLN3* gene is predicted to encode a hydrophobic six transmembrane domain protein (Ratajczak et al., 2014) and is conserved in many species including yeast and *Drosophila* but its function is unclear despite more than 20 years of study. Various studies of *CLN3* in different cell lines and models have suggested roles in regulation of lysosomal pH, anterograde and retrograde post-Golgi trafficking, autophagy, endocytosis, apoptosis, oxidative stress responses or Notch and JNK signalling (Tuxworth et al., 2011, Tuxworth et al., 2009 and reviewed in Carcel-Trullols et al., 2015). The expression pattern of the *CLN3* gene in mice is known from a combination of *in situ* hybridization studies and a knock-in reporter of gene expression (Ding et al., 2011, Eliason et al., 2007). The knock-in mouse, in particular, demonstrated an expression pattern in the CNS predominantly in the later stages of embryonic development and persisting in post-natal development. Interestingly, strong expression from early stages of development in the endothelia of the brain was maintained into adult life (Eliason et al., 2007). *CLN3* expression was also detected in endothelia in other organs, in epithelia and strongly in the renal tubules, where its expression is regulated by osmolality (Stein et al., 2010). Taken together, these data suggest an important role for *CLN3* in epithelia but since the reporter used was a nuclear-localized β-galactosidase, the sub-cellular localization of CLN3 protein in polarized epithelial cells could not be determined. CLN3 is considered primarily a lysosomal protein, based on numerous studies in cell culture with epitope-tagged or fluorescent fusion proteins (reviewed in Phillips et al., 2005) and it has been identified in lysosomal membranes by proteomics (Chapel et al., 2013). Studies of mammalian CLN3 localization have generally relied on overexpression; one of the few studies to detect endogenous CLN3 indicated a mitochondrial localization in Müller glia of the mouse retina and in the inner segments of photoreceptors (Katz et al., 1997). Knock-in approaches have been used in yeast to avoid overexpression artefacts and reveal CLN3 can be found in the Golgi (Kama et al., 2011) or at the vacuole (the yeast lysosomal equivalent) with sub-cellular localization regulated by intracellular pH (Wolfe et al., 2011). In *Dictyostelium*, GFP-CLN3 expressed at low levels localizes to the osmoregulatory contractile vacuole and other endocytic vesicles (Huber et al., 2014) but knock-in approaches have not been used to date to study CLN3 in a species with a complex nervous system.

The lack of reagents for reliable detection of endogenous protein localization *in vivo* for either CLN7 or CLN3 has hampered the search for their functions. To overcome these limitations, we used recombineering and CRISPR/Cas9 genome editing to generate seamless knock-in YFP fusions of *CLN7* and *CLN3* to report gene expression and protein localization in *Drosophila*. We show that *CLN7* is strongly expressed in glial cells in the CNS but largely absent from neurons other than in the developing visual system. *CLN3* is also expressed in glia and very strongly in Malpighian tubules, the insect organ orthologous to the kidney. Unexpectedly, CLN3 protein in tubules is localized to the apical domain and also to mitochondria. These findings alter our ideas of *CLN* gene function and suggest new possibilities for the causes of NCL disease.

## 2 Materials and methods

### 2.1 *Drosophila* stocks and husbandry

Flies were maintained in vials on standard agar/yeast-based media at 25 °C and 12-h light/dark cycle except during the genome editing and recombineering procedures where semi-defined medium was used to boost egg laying (recipes available from Bloomington Stock Center website). The control line used for all experiments was an isogenic *w*^1118^ strain (BL 6326).

### 2.2 CRISPR/Cas9-mediated HDR of *CLN7*

**Fig. 1:**
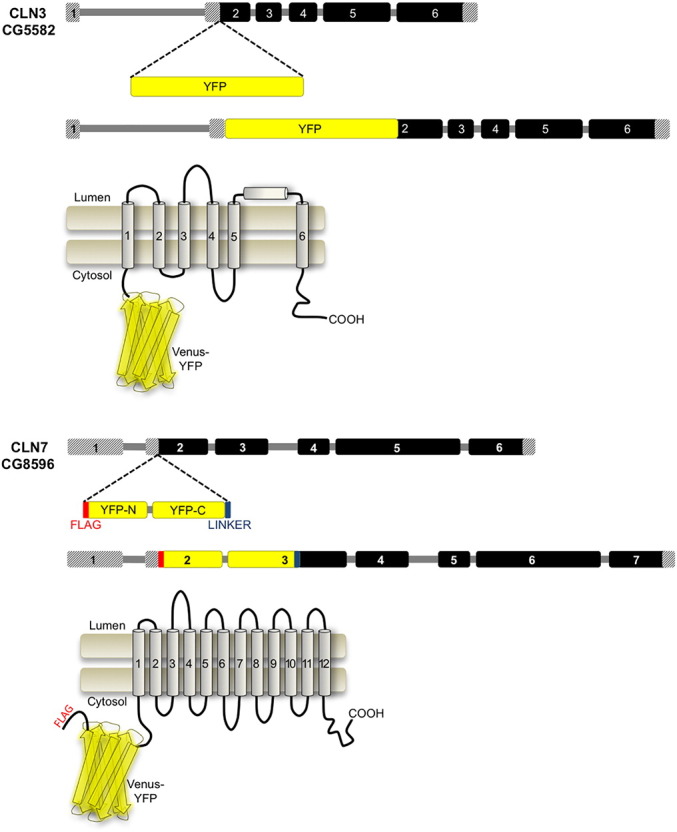
Strategy to generate YFP-*CLN3* and YFP-*CLN7* knock-ins. The Venus variant of YFP was inserted after the ATG start codon of *CLN7* or *CLN3* to generate N-terminal fusion proteins. For *CLN3*, a BAC containing the *CLN3* locus and surrounding sequences was modified by seamless recombineering to include YFP after the ATG start codon then the BAC inserted into a landing site on chromosome II by ϕC31-mediated recombination. For *CLN7*, CRISPR/Cas9-mediated homologous recombination was used to insert YFP into the *CLN7* locus. A single FLAG-tag was included at the N-terminus of YFP and a short flexible linker between YFP and *CLN7*. An artificial intron from the *white* gene was included in the YFP sequence. Based on predicted topologies, YFP should be on the cytosolic side for both proteins. CLN3 is likely to exist as a dimer.

A gRNA was selected using fly CRISPR Optimal Target Finder (tools.flycrispr.molbio.wisc.edu/targetFinder). Oligos corresponding to the gRNA sequence were cloned into BbsI digested pCFD3 (Port et al., 2014) and confirmed by Sanger sequencing. Templates for homology-directed repair was designed to incorporate the *Drosophila* codon-optimised Venus-YFP sequence immediately 3′ of the *CLN7* start codon followed by 15 bp encoding GGAGG as a linker. 500 bp of *CLN7* sequence either side of the site of the Cas9 digestion site were included as homology arms. In one version, an artificial intron from the *white* gene was incorporated into the YFP sequence to reduce the size of the expanded YFP-containing first exon (Fig. 1). qPCR suggested both intron-containing and intron-less genes were expressed at similar levels. Constructs were synthesized by GenScript. The gRNA and HDR template were co-injected as supercoiled plasmids into *vasa::Cas9* embryos (BL51323) at a mixed ratio of 250:750 μg/μl respectively at the Department of Genetics, University of Cambridge. Injected flies were crossed to a third chromosome balancer and successful incorporation of the YFP sequence into CG8596 detected by PCR from gDNA after mating. Germline transmission was followed after mating by single fly gDNA PCR.

### 2.3 Recombineering of *CLN3*

The Venus variant of YFP was incorporated immediately downstream of the ATG start codon of the *CLN3* locus by recombineering of pBAC CH322-133O15 (pacmanfly.org; (Venken et al., 2009)). A two-step recombineering procedure was used essentially as described for the seamless incorporation of tags into *C. elegans* cosmids but modified in this case for the use of *Drosophila* BACs (Dolphin and Hope, 2006). 75 bp of *CLN3* sequence either side of the integration site were used for insertion of the TetR selection cassette into *CLN3* and for the subsequent swap of TetR for YFP (Fig. 1A). Clones were validated by Sanger sequencing and transgenic flies were generated by ϕC31-mediated recombination into the attP40 site by BestGene. Successful transformants were identified by PCR after mating to balancer. Standard crossing schemes were used to generate stable stocks.

### 2.4 Immunofluorescence microscopy

Wandering third instar larvae CNS, imaginal discs, salivary glands and Malpighian tubules were dissected in HL3.1 buffer solution (Feng et al., 2004) then fixed in 4% methanol-free EM-grade formaldehyde (PolySciences) in HL3.1 for 30 min on ice. Salivary glands and Malpighian tubules samples were subsequently permeabilised for 10 min and all washing steps were performed in PBS with 0.1% Tween-20 or PBS with 0.3% Triton X-100 for all steps with CNS and discs. Samples were blocked in 1% BSA in PBS/Tween or PBS/Triton for 30–60 min at room temperature. Samples were incubated with primary antibodies diluted in block overnight at 4 °C, washed several times then incubated with the appropriate secondary antibodies in block for 2 h at room temperature. After a repeat of the washing regime, samples were mounted in Prolong Gold (Invitrogen) or VectaShield (Vector Laboratories). Details of antibodies used, suppliers and dilutions are provided in Supplementary materials. DNA was visualized by incubation with To-Pro 3 (Invitrogen). Images were acquired on a Zeiss LSM780 or a LSM510 Meta confocal microscopes.

### 2.5 Immunoelectron microscopy

Immunoelectron microscopy of Malpighian tubules was carried out after the method developed by Tokuyasu (Tokuyasu, 1986). Tubules were fixed in 4% EM-grade formaldehyde for 1 h before cryoprotection by incubating in 2.3 M sucrose overnight. Small pieces of tubule were mounted on cryopins and cryofixed by plunging into liquid nitrogen. The cryopins were stored under liquid nitrogen until required for sectioning.

Sections (90 nm thick) were cut with glass knives at ‐100 °C using a Leica cryo-ultramicrotome. Sections were collected on droplets of a mixture of 2.3 M sucrose, 2% methyl cellulose and transferred to pioloform-coated 150 hexagonal mesh Ni Grids. The grids were floated on standard buffer consisting of PBS containing 1% BSA (Jackson Laboratories) and 0.1% sodium azide for at least 1 h. Grids were incubated with PBS containing 50 mM glycine for 15 min to block any remaining fixative and then washed 3 × 5 mins by incubating over droplets of standard buffer. Each grid was then incubated on an individual droplet of primary rabbit anti-GFP (Invitrogen) diluted 1:200 in standard buffer. Controls were incubated on standard buffer only. Excess primary antibody was removed by washing the grids thoroughly by placing on droplets of standard buffer (6 droplets, 5 mins each) before incubation on droplets of the gold-conjugated anti-rabbit antibody (Jackson labs) diluted 1:100 in standard buffer. Grids were washed as before in standard buffer before a final wash in PBS without any additives and fixed using PBS containing 2% glutaraldehyde for 5 mins. The grids were then washed over droplets of distilled water before contrasting by placing on droplets of a solution containing nine parts 2% methylcellulose to one part 3% uranyl acetate for 20 mins. The grids were drained by blotting against wedges of filter paper and dried before examination. Sections were examined using a Tecnai T12 electron microscope operated at 100 kV and images were captured using an AMT Camera.

### 2.6 Live imaging of Malpighian tubules

Malpighian tubules from wandering third instar larvae were dissected in HL3.1 containing 1:10,000 Lysotracker Red (Invitrogen) and imaged immediately on bridge slides on an upright Zeiss LSM510 confocal microscope. Laser levels were kept low to reduce the possibility of photoconversion. Only the upper portions of the tubule could be imaged due to poor laser penetration into deeper regions.

### 2.7 Figure preparation

Confocal images of the larval CNS and dissected larval body wall muscles were stitched post-acquisition on the Zeiss 780 confocal microscope using Zeiss Zen software when tiling was required. Single XY images from z-stacks were exported to TIFF from Zen or Volocity software (Perkin Elmer). z-stacks were rendered in Zen and Volocity, snapshots taken and exported as TIFF files. Note the zoom of rendered images may vary from the XY images and so no scale bar is included. Final figures were prepared in Photoshop CS6. The resolution of the TEM montage was reduced by resampling in the “Image Size” menu to necessarily reduce the file size.

## 3 Results

Identifying the expression patterns and sub-cellular localization of NCL proteins will help elucidate their functions and further our understanding of cellular pathology in the NCLs. We raised antibodies to both *Drosophila* CLN7 (unpublished) and CLN3 proteins (Tuxworth et al., 2009) but both proved poorly antigenic. Our anti-CLN3 antisera does recognize endogenous CLN3 in fixed samples but only in tissues where it is highly expressed (Fig. 6) and it shows additional non-specific reactivity. To overcome these problems, we used two different strategies to generate knock-ins: CRISPR/Cas9-mediated homology-directed repair to make a YFP-*CLN7* knock-in and seamless recombineering of a BAC to make a YFP-*CLN3* knock-in (Fig. 1 and methods). This allowed us to use highly-specific anti-GFP antibodies to detect proteins at endogenous levels in fixed samples, use live imaging and, importantly, meant the proteins were not overexpressed. This avoided a potential confounding issue for membrane proteins whose localization may change when overexpressed.

### 3.1 *CLN7* and *CLN3* are expressed in glia forming the blood-brain-barrier

**Fig. 2:**
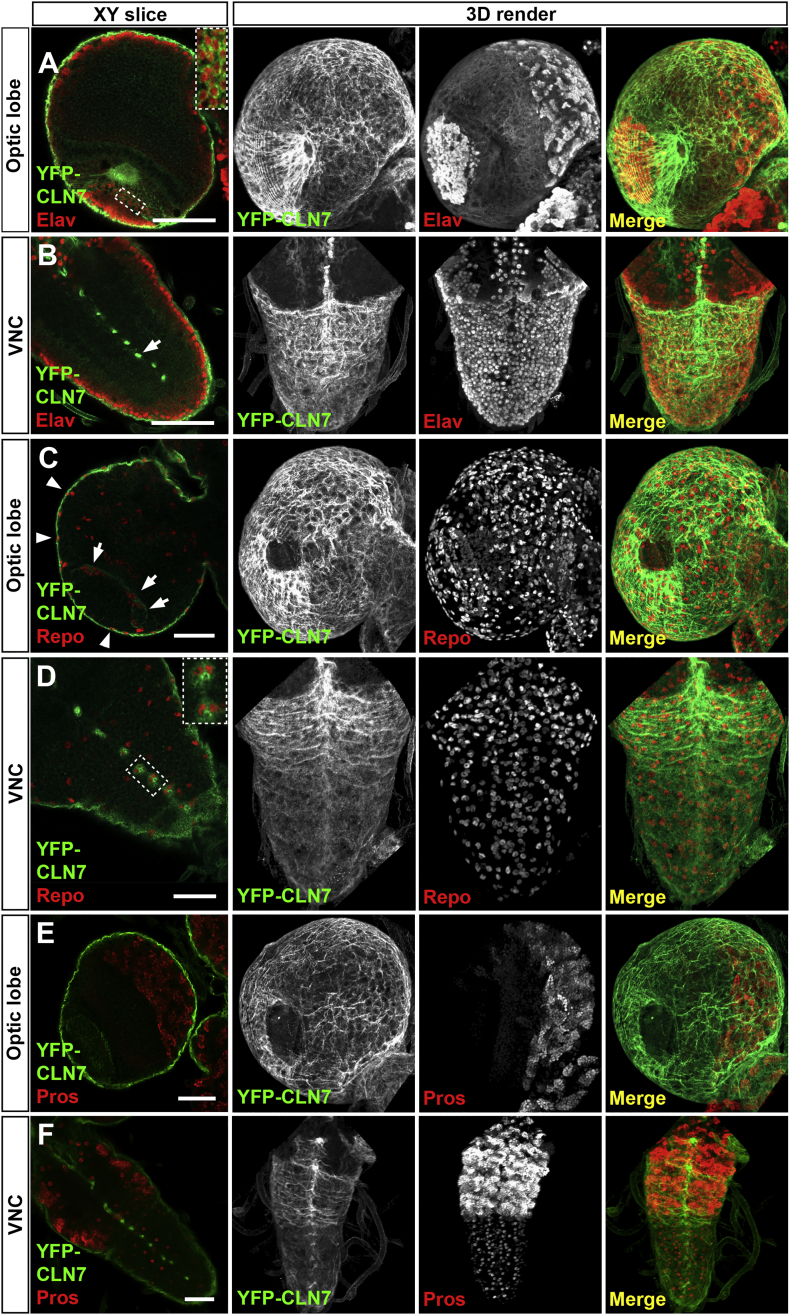
*CLN7* is expressed in subsets of glia in the CNS. Fixed CNS from late YFP-*CLN7* larvae imaged by confocal microscopy. Single XY optical slices and the corresponding 3D rendered volumes of the optic lobe and ventral nerve chord (VNC) are shown. Note the 3D rendered images have been rotated in some instances. YFP-CLN7 localization detected with anti-GFP is shown in green in each case. A, B: *CLN7* is strongly expressed in the surface glia ensheathing the CNS with some expression in the optic lobe. There is overlap between *CLN7* expression and the post-mitotic neuronal marker, anti-Elav (red) in lamina neurons in the optic lobe (enlargement shown in box in A). Strong expression in the midline of the VNC (arrow in B.) does not co-localize with anti-Elav. C, D: The pan-glial marker anti-Repo (red) shows YFP-CLN7 co-localization with Repo-positive nuclei around the extreme periphery of the CNS in the surface glia (arrowheads in C), in one part of the optic lobe (arrows in C) and in channel glia in the VNC (enlargement shown in box in D). The 3D renders show YFP-CLN7 in the surface glia covering the surface of the CNS with each cell encircling a Repo-positive glial nucleus. E,F. No overlap between YFP-CLN7 localization and anti-Prospero, a marker of neural and glial precursors. Scale bar = 50 μm.

**Fig. 3:**
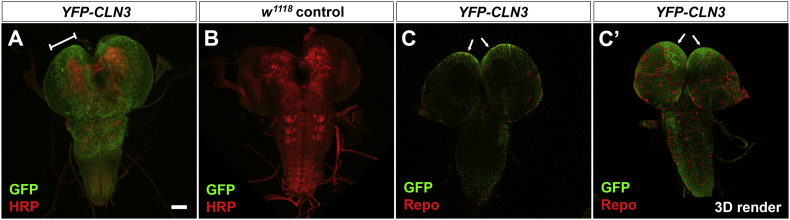
*CLN3* is expressed in surface glia in the CNS. Fixed CNS from late YFP-*CLN3* larvae imaged by confocal microscopy. A,B: YFP-CLN3 localization was detected with anti-GFP (green) and neuronal plasma membranes with anti-HRP (red) in the CNS from YFP-*CLN3* transgenic flies (A) or *w*^1118^ control flies (B). YFP-*CLN3* is expressed at low levels in the surface glia surrounding the CNS. anti-GFP reactivity is absent in the control images captured and processed identically. Expression is stronger in cells over the central brain region (bracketed in A). C: YFP-CLN3 (green) co-localizes with the pan-Repo nuclear marker anti-Repo (red) in surface glia. Higher expression of *CLN3* in glia over the central brain region is indicated by arrows. C,C': single XY optical slice and C’ 3D rendered volume. Scale bar = 100 μm.

**Fig. 4:**
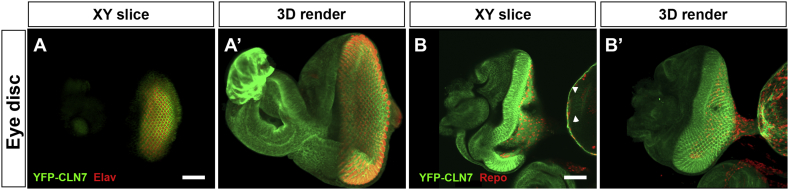
*CLN7* is expressed in neurons and glia in the developing visual system. Fixed eye imaginal discs from YFP-*CLN7* larvae stained with anti-GFP (green). A,A’: anti-Elav (red) marks neuronal photoreceptors. B,B’ anti-Repo (red) marks glia (B,B’). A and B show single XY sections; A’ and B’ show 3D renders of z-stacks. Note the expression of *CLN7* in both neurons and glia and the distribution of CLN7 along photoreceptor projects into the visual centres of the brain in B and B’. Scale bar = 50 μm.

Since pathology is largely restricted to the CNS in the NCLs, we examined expression of the CLN7 and CLN3 proteins in the mature larval nervous system. The *Drosophila* larval nervous system is composed of two major regions, the optic lobes and ventral nerve cord. Expression profiling has previously indicated only low levels of expression of *CLN3* and *CLN7* transcripts in the late larval nervous system (www.flyAtlas.org: Chintapalli et al., 2007; *CLN7* = CG8596; *CLN3* = CG5582). We were surprised to find that this is because both CLN7 and CLN3 proteins are largely restricted to subsets of glia in the CNS (Fig. 2, Fig. 3, Fig. 4) with expression only in specific neurons. Although *CLN7* is expressed predominantly within glia we do observe expression of *CLN7* in neurons of the developing visual system, both in the larval eye disc and in the optic lobe of the brain (Fig. 2, Fig. 4), consistent with the early degeneration of the visual system in NCL patients.

Both proteins are expressed in the peripheral surface glia that form the blood-brain-barrier (BBB) in insects (Limmer et al., 2014, Stork et al., 2008). This is particularly striking for *CLN7*, which is expressed at higher levels in surface glia around the entire CNS (Fig. 2). YFP-CLN7 staining overlaps with anti-Repo staining, which identifies all glia with the exception of midline glia, and does not overlap with anti-Elav staining (post-mitotic neurons) or anti-Prospero (neural and glial precursors). Optical sectioning the larval brain and ventral nerve cords from YFP-*CLN7* expressing larvae (Fig. 2. XY slice) followed by rendering of the sections into 3D clearly shows *CLN7* ensheathing the optic lobes and the ventral nerve cord (Fig. 2). We found YFP-*CLN7* was expressed in a large number of cells with small, oblong nuclei on the apical side of the brain that are the perineurial glia (Fig. 2C, arrowheads), but was absent from the more basal sub-perineurial glia which have characteristic larger nuclei (Awasaki et al., 2008). *CLN7* is also expressed in cells that appear to be the channel glia in the ventral nerve chord (Fig. 2B and D, arrow and inset box respectively).

As an alternative approach to reveal the sites of *CLN7* expression we generated a promoter fusion construct in which approximately 1.8 kb of gene regulatory sequence upstream of the *CLN7* start codon was used to drive expression of the nuclear RedStinger reporter (Barolo et al., 2004). RedStinger expression pattern mirrors that of the YFP-*CLN7* knock-in although it is expressed additionally in some other glia in the CNS which were not YFP-positive (see Supplementary Fig. S1). Both the YFP-*CLN7* knock-in and *CLN7*::RedStinger reporter reveal that *CLN7* expression is found mostly within glia and a small subset of neurons. Surprisingly, neither method to visualize *CLN7* expression revealed any expression in central neurons of the CNS.

YFP-*CLN3* is expressed at very low levels in the CNS but high affinity anti-GFP antibodies detect expression in glia at the surface of the brain. *CLN3* expression is clearly higher in regions overlying the central brain in comparison to the optic lobes (Fig. 3). These expression patterns correlate with transcriptome data from surface glia which showed a strong enrichment of *CLN7* (CG8596) and lower expression of *CLN3* (CG5582) (DeSalvo et al., 2014).

### 3.2 *CLN7* is expressed in the developing visual system

Early retinal pathology is common to all forms of NCL and is exhibited by a *CLN7* mutant mouse (Jankowiak et al., 2016). It is interesting, therefore, that *CLN7* is expressed in the developing visual system of *Drosophila* larvae, both in the imaginal eye discs that give rise to structures of the eye and in the optic lobes of the CNS into which the photoreceptors project (Fig. 4). In the discs, YFP-CLN7 protein is seen in the Elav-positive neurons of each ommatidium (Fig. 4A, A’) and in glia stained with Repo (Fig. 4B, B’). In the optic lobe, YFP-CLN7 can be seen in the lamina neurons and neuronal projections (Figs. 2A and 4B, indicated by arrowheads in both).

### 3.3 *CLN7* is enriched at the post-synaptic density

**Fig. 5:**
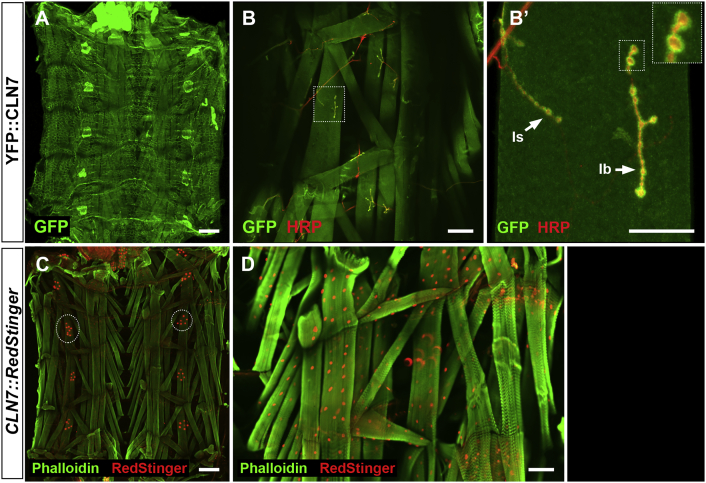
CLN7 is recruited to the post-synaptic density at the neuromuscular junction. Late-stage YFP-*CLN7* larvae were dissected and fixed to observe the body-wall muscles and innervations. A: *CLN7* (anti-GFP, green) is expressed in all body wall muscles, oenoctyes and in the overlying epidermis. B: YFP-CLN7 protein is recruited to the post-synaptic density at all neuromuscular junctions. YFP-CLN7 is detected with anti-GFP (green) and neuronal membranes with anti-HRP (red). Muscle IV is boxed. B’ Higher magnification of B. CLN7 is recruited to both type Is and Ib neuromuscular junctions (arrows). Two type Ib boutons are further magnified in the inset box. C, D: Strong expression of *CLN7* in oenocytes (circled in C) and all body-wall muscles (D) is confirmed with the nuclear RedStinger promoter fusion (red). F-actin is visualized with Alexa-488 phalloidin (green). Scale bar = 250 μm (A and C); 100 μm (B and D); 50 μm (B’).

We have identified roles for *CLN7* in the development of the larval neuromuscular junction (NMJ), a model for synaptic assembly and neural development (O'Hare, Mohammed, Tuxworth and Tear, unpublished). Since *CLN7* appears not to be expressed in the motor neurons that innervate the NMJ (Fig. 2), we examined whether the protein is expressed in the post-synaptic muscle tissue. Both the YFP-*CLN7* knock-in and the *CLN7*::RedStinger reporter show expression in all body wall muscles in wandering third instar larvae (Fig. 5A, D), in segmentally repeated clusters of oenocytes (Fig. 5A and circled in Fig. 5C) and in the overlying epithelial cells. Importantly, YFP-CLN7 is concentrated at the NMJ in each of the muscles forming clusters around each swelling (bouton) of the pre-synaptic membrane (Fig. 5B, B’). The anti-GFP staining is clearly separable from the pre-synaptic membrane visualized with anti-HRP (red in Fig. 5B, B’). This is true for both the type Ib and Is junctions and indicates recruitment to, or concentration at, the post-synaptic density.

### 3.4 *CLN3* localizes to the apical domain of tubules

**Fig. 6:**
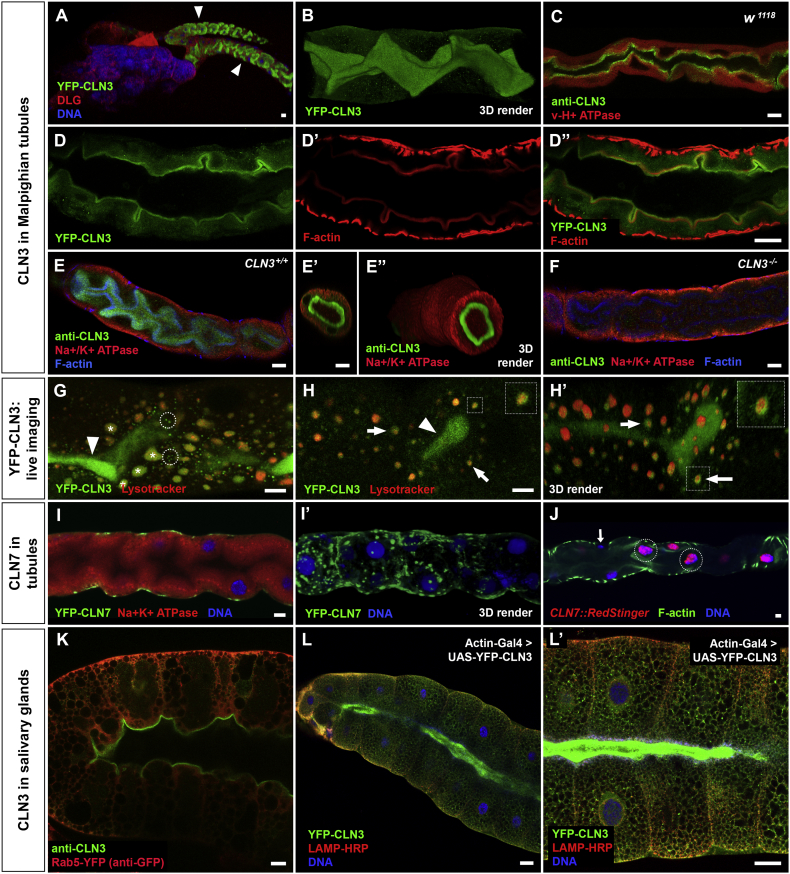
CLN3 in Malpighian tubules localizes to the apical domain. *CLN3* is highly expressed in Malpighian tubules (the insect excretory and detoxification organ). A: low magnification image of the junction between gut and tubules (arrowheads indicate each tubule) from a YFP-*CLN3* larva stained to visualize septate junctions (anti-DLG, red), DNA (blue) and YFP-*CLN3* expression (anti-GFP, green). Note the localization of YFP-CLN3 to the convoluted lumen of the tubules. B: 3D render of a z-stack through a YFP-*CLN3* tubule stained with anti-GFP to show the localization of YFP-CLN3 to the lumen. C: single XY slice through tubule from a v-H^+^ ATPase GFP protein trap line stained with anti-CLN3 (green) and anti-GFP (red). v-H^+^ ATPase is excluded from the apical domain. This confirms the apical localization of endogenous CLN3 and demonstrates that the YFP fusion to CLN3 does not affect localization. D: a co-stain of anti-GFP (green, D) with Alexa-546 phalloidin to visualize F-actin in tubules (D’, red). The merged image (D”) regions shows YFP-CLN3 more apical than the band of F-actin running underneath and parallel to the lumen. E: anti-CLN3 staining of wild-type tubules confirms the localization of endogenous CLN3 protein to the apical domain abutting the lumen. Tubules are triple-stained with anti-CLN3 (green), anti-Na^+^/K^+^ ATPase (red) and phalloidin (blue). E’ and E” show a single XZ optical section and a 3D render of the volume of the same tubule as in E. For clarity, F-actin is not shown. Note the clear separation between apical (luminal) CLN3 and basal Na^+^/K^+^ ATPase. F: tubule from a *CLN3*^−/−^ null larva stained and imaged identically to (E) to show the specificity of the anti-CLN3 antibody. G&H: Live imaging of YFP-CLN3 (green) in tubules counterstained with lysotracker (red) to mark low-pH endosomes and lysosomes. G: A single XY optical section from the upper part of a tubule reveals small foci of YFP-CLN3 present in unidentified vesicles (circled). The large lysotracker-positive vesicles are autofluorescent (asterisks). H, H’: a single XY section (H) from the upper part of a tubule shows YFP-CLN3 apparently localized to the membrane of some lysotracker-positive vesicles (arrows; boxed vesicle is enlarged in inset). The accompanying 3D–rendered image shows the membrane localization more clearly (arrows; boxed vesicle is enlarged in inset). Note the convoluted lumen with high levels of YFP-CLN3 also enters the optical sections of (G) and (H) in places (arrowheads). I&J: *CLN7* is also expressed in tubules. A single XY optical slice (I) and a 3D render (I’) of a YFP-*CLN7* tubule stained with anti-GFP (green) and anti-Na^+^/K^+^ ATPase (red) reveal CLN7 is largely localized in large, peripheral vesicles likely to be lysosomes. J: expression of *CLN7* only in the principal cells of the tubule is confirmed with the nuclear RedStinger promoter fusion (red). Arrows point to stellate cells not expressing YFP-*CLN7*. K&L: CLN3 is also localized to the apical domain of salivary glands, which are also single cell tubules. K: anti-CLN3 staining (green) from a larva expressing YFP-Rab5 as a counterstain (red, detected with anti-GFP). L&L’: YFP-tagged CLN3 expressed from a UAS-transgene. Despite overexpression, YFP-CLN3 remains apical (green). LAMP-HRP and DNA stains show morphology (red and blue). L’ is a higher magnification image of a central section of L. Scale bars = 5 μm (A-J); 20 μm (I); 50 μm (L&L’).

**Fig. 7:**
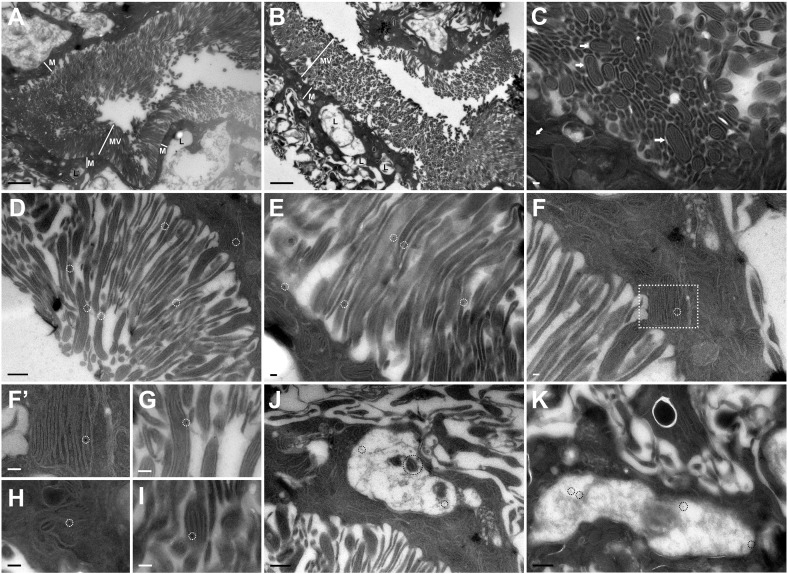
ImmunoEM localizes CLN3 to microvilli, mitochondria and lysosomes. ImmunoEM staining of YFP-*CLN3* transgenic tubules from late larvae were stained with rabbit anti-GFP and gold-conjugated anti-rabbit antibodies. A–C were stained without primary antibody and no gold particles are visible, demonstrating the specificity of the secondary antibody. A–C: The morphology of control tubules reveal important features of the insect Malpighian tubule. A,B: Microvilli (MV) protrude into the tubule lumen; many mitochondria are visible immediately below the microvilli (M); large vacuoles reported previously to be lysosomes are visible (L; Pal and Kumar, 2013). C: Numerous mitochondria are present within and at the base of microvilli (arrows). D&E: Gold particles corresponding to YFP-CLN3 are localized to microvilli, likely in the membrane (white circles). F: A gold particle within a mitochondrion (circle, magnified in F’). G–I: Higher magnification examples gold particles found in microvilli (G) and within mitochondria (H&I). J&K: Gold particles within large lysosome-like vesicles (black circles). Note also the presence of a mitochondrion within a lysosome (J, large black circle). Scale bars = 2 μm (A,B); 500 nm (D,J,K); 100 nm (E–I), 50 nm (F’).

FlyAtlas (Chintapalli et al., 2007) indicates high levels of *CLN3* transcription in Malpighian tubules, the insect organ orthologous to the kidney. We fixed and stained tubules from YFP-*CLN3* expressing animals with anti-GFP and wild-type tubules with anti-CLN3 to visualize *CLN3* expression (Fig. 6A–E,). In both cases we saw strong expression of *CLN3*, as predicted by the expression profiling, which was absent in *CLN3* homozygous mutant tubules (Fig. 6F). However, we were surprised to find that the majority of CLN3 protein localizes to the extreme apical domain that forms the lumen of the tubule (Fig. 6A–E). An unrelated GFP protein trap inserted in the Vha55 gene encoding a vacuolar H^+^ ATPase does not localize to the apical domain and is clearly separable from CLN3 protein (Fig. 6C). Tubules were co-stained with antibodies to the Na^+^/K^+^ ATPase transporter that resides in the basal membrane. The apical CLN3 and basal Na^+^/K^+^ ATPase were clearly separated spatially (Fig. 6E). The Malpighian tubules have a thick network of actin-rich microvilli protruding into the lumen. Interestingly, staining YFP-*CLN3* tubules with anti-GFP and phalloidin to visualize the F-actin indicates that CLN3 is found more apical than the predominant F-actin band: the two signals are clearly resolvable by standard confocal microscopy (Fig. 6D). This suggested a potential localization of CLN3 to the tips of the microvilli. The low-affinity anti-CLN3 shows some non-specific reactivity hence, only the strongest CLN3 signal can be detected unambiguously. However, the highly specific anti-GFP reveals some additional CLN3 protein in tubules, especially in small foci (Fig. 6B, D). To gain further insight into this potential additional localization, we dissected YFP-*CLN3* expressing tubules, stained then briefly with the vital low pH vesicle marker, lysotracker, and imaged them by confocal microscopy without fixation. Single optical sections taken near the upper surface of the tubules reveals large, autofluorescent vesicles positive for lysotracker that are likely to be lysosomes (Fig. 6G, marked with asterisks; compare also with TEM in Fig. 7 and other reports of the ultrastructure of lysosomes of insect Malpighian tubules (Pal and Kumar, 2013)). Again, small foci of YFP-CLN3 were visible (Fig. 6G, circled) likely corresponding to the small foci seen in fixed samples (cf*.* Fig. 6B and D). Additionally, some YFP-CLN3 appeared to be localized to the membrane of the lysotracker-positive vesicles (Fig. 6H and H’), consistent with the multi-span transmembrane nature of the CLN3 protein.

Salivary glands in *Drosophila* are also single cell-thick tubules. Interestingly, the anti-CLN3 antibody also reveals strong localization of endogenous CLN3 to the apical side of salivary gland cells adjacent to the central lumen (Fig. 6K). Moreover, a YFP-tagged CLN3 protein remains predominantly localized adjacent to the lumen even under overexpression conditions (Fig. 6L, L’).

### 3.5 CLN3 localizes to microvilli, mitochondria and lysosomes in tubules

To gain increased resolution of this intracellular expression we used immuno-electron microscopy of frozen sections of Malpighian tubules. The microvilli in insect Malpighian tubules are much longer than vertebrate microvilli and possess mitochondria along their length (Ryerse, 1979). Mitochondria are also present at the base of microvilli with large, irregularly-shaped lysosomes present more basally corresponding to the large lysotracker-positive vesicles seen in the live imaging (Fig. 6G,H). Using anti-GFP in YFP-*CLN3* tubules with a gold-labelled secondary antibody, we detected CLN3 along microvilli, usually close to the membrane (Fig. 7D,E,J). We were surprised to see CLN3 localized within mitochondria, most clearly in the mitochondrial-rich area at the base of microvilli (Fig. 7F,G,I,J). Since we used a primary and secondary antibody to detect the CLN3 protein, the location of the YFP molecule could be anywhere within an approximately 20 nm radius of each gold particle but despite this, several gold particles were comfortably within mitochondria. It seems likely that the intense apical YFP fluorescence seen in the lumen of tubules by live imaging corresponds to YFP-CLN3 accumulation in microvilli and the small more basally located foci to YFP-CLN3 in mitochondria. We also detected occasional localization of YFP-CLN3 to the large vacuolar organelles, which we deduce are lysosomes based on the large, lysotracker-positive vesicles seen in the live imaging (Fig. 6 G,H) and on their highly similar appearance to lysosomes in other ultrastructural studies of insect Malpighian tubules (Pal and Kumar, 2013). The lysosomes include one apparently containing a mitochondrion, possibly during mitophagy (Fig. 7J).

*CLN7* is also expressed in the principal cells of Malpighian tubules where it is predominantly localized to large peripheral vesicles likely to be lysosomes (Fig. 6I). The RedStinger promoter fusion indicates *CLN7* is not expressed in stellate cells (Fig. 6J, *CLN7* + principal cell nuclei indicated with circles and *CLN7* − stellate cells nuclei with arrows).

## 4 Discussion

We have used genome engineering and recombineering to generate knock-in constructs to generate YFP tagged CLN7 and CLN3 proteins expressed from their endogenous loci. We have used these lines to overcome the poor antigenicity of the CLN7 and CLN3 proteins and allow the investigation of their cellular expression and sub-cellular localization. Surprisingly, we found that both *CLN7* and *CLN3* have restricted expression in the CNS: *CLN7* is expressed in neurons in the developing visual system, but not widely in neurons elsewhere, and *CLN3* is absent from neurons. Instead, in the CNS both CLN7 and CLN3 are primarily glial proteins. Unexpectedly, we find that CLN3 is additionally localized at the apical domain of tubules and in mitochondria.

There is always the possibility that a fluorescent fusion protein might not fold correctly, thus affecting its localization. We believe this to be unlikely in this study since YFP-CLN3 localization matches the endogenous CLN3 protein where we are able to visualize it. We attempted to reduce the possibility of mis-folding as far as possible by inserting YFP at the extreme N-terminus of both *CLN7* and *CLN3* coding sequences. Many groups have used N-terminal fusions of GFP to CLN3 in a variety of models without retention of the protein in the ER (Codlin and Mole, 2009, Huber et al., 2014, Oetjen et al., 2016) and GFP-CLN3 is functional in both yeast (Codlin and Mole, 2009) and *Dictyostelium* (Huber et al., 2014). In contrast, we and others have shown that truncated forms of CLN3—or adding epitope tags or GFP to the C-terminus—does lead to ER retention and a very different sub-cellular staining pattern than that we see here (example studies include Haskell et al., 1999, Jarvela et al., 1999, Tuxworth et al., 2009). In the case of YFP-CLN7, we have no antibody to detect endogenous CLN7 protein and there have been few comparable studies. However, we can rescue the neurodevelopmental phenotype of *CLN7* mutant animals by re-expressing a YFP-*CLN7* construct (O'Hare, Mohammed, Tuxworth and Tear, in prep). This indicates YFP-CLN7 is functional so must be folded and localized correctly.

### 4.1 Expression of *CLN7* and *CLN3* in glia

We find that *CLN7* and *CLN3* are largely restricted to glia in the *Drosophila* CNS but this contrasts with the mammalian CNS where both genes are also expressed widely. Indeed, *CLN7* transcripts are several-fold higher in neurons than in glia in the rat CNS (Sharifi et al., 2010). One possibility is *Drosophila* reflects an ancestral expression pattern in glia that has been expanded to expression throughout the CNS in vertebrates. Autofluorescent storage material accumulates widely in CNS of human NCL patients but is not detectable in mutant *CLN3* or *CLN7* flies (O'Hare and Tuxworth, unpublished) consistent with the restricted expression of the genes. It is notable that activation of glial cells is the earliest detectable event in animal models of various NCLs (Cooper et al., 2015, Kay et al., 2006, Oswald et al., 2005, Pontikis et al., 2004) but the triggers for glia activation in NCL and the roles that glial cells may play in the disease process are not well understood. Studying *CLN3* and *CLN7* function in glia in *Drosophila* may give some insight into these early events in NCL pathology.

We could not detect *CLN3* expression in the visual system at the third instar larval stage of development but *CLN7* is expressed in neurons and glia at this stage. Atrophy of the visual system is a common feature of all of the childhood-onset forms of NCL and is seen in *CLN7* mutant mice (Jankowiak et al., 2016); again, this may reflect a conserved ancestral role for *CLN7* and it will be interesting to determine whether *CLN7* mutant flies suffer similar visual pathology.

### 4.2 Potential functions for *CLN7* in the Blood-Brain-Barrier

We found *CLN7* to be primarily expressed in the surface glia, which form the BBB in insects (Stork et al., 2008). The surface glia comprise two different types: the apical perineurial glia, which express numerous solute transporters, and the underlying sub-perineurial glia, which form occlusive tight-junctions, analogous to the endothelial tight junctions in the vertebrate BBB. CLN7 appears to be restricted to the apical perineurial glia, in keeping with its likely role as a solute transporter. Interestingly, YFP-CLN7 appears to be localized predominantly at, or near the plasma membrane in these glia. We did not expect to see this localization at the outset of the study because human and mouse CLN7 are predominantly late-endosomal and lysosomal proteins in cultured cells (Sharifi et al., 2010, Steenhuis et al., 2010) and has been identified in lysosomal membranes by proteomics (Chapel et al., 2013). However, CLN7 in Malpighian tubule principal cells was predominantly localized in large vesicles likely to be lysosomes. Both the surface glia and Malpighian tubules regulate solute transport—across the BBB and gut respectively—so it seems likely that CLN7 contributes to solute transport in both cell types. One possibility is that the transporters are localized differently in the glia and the tubules or possibly that the thin, stretched nature of the perineurial cells falsely gives the impression of plasma membrane localization.

Both principal and stellate cells express numerous solute transporters for osmoregulation, excretion and detoxification but the absence of CLN7 from stellate cells indicates it is unlikely to be involved in chloride transport. Interestingly, the surface glia also contribute to regulation of organismal growth indirectly through secretion of a soluble insulin receptor antagonist, SDR (Okamoto et al., 2013). Reduced expression of SDR results in increased insulin signalling and increased growth of the fly, a phenotype that is also present in *CLN7* mutants (O'Hare, Mohammed, Tuxworth and Tear, unpublished) so it will be of interest to determine whether SDR secretion is affected by *CLN7* loss.

### 4.3 *CLN3* and other NCL gene expression in glia

*CLN3* is also expressed in surface glia but the expression is low and is not uniform in all surface glia of the larval CNS (Fig. 3). Expression appears to be stronger in the region overlying the central brain (Fig. 3, bracketed section) and lower or absent in glia overlying the optic lobes. Transcriptome profiling of surface glia confirms *CLN3* expression and while it is not clear what role *CLN3* might play in these glia, it is interesting to note that disruption to the BBB has been reported in human Juvenile NCL and in *CLN3* mutant mice and that cultured *CLN3*^*−/−*^ endothelial cells show disruption to membrane microdomains and trafficking (Tecedor et al., 2013).

### 4.4 *CLN3* localization to the apical domains of tubules

CLN3 is widely reported to be a lysosomal protein. However, the lack of high-affinity antibodies for endogenous CLN3 means almost all studies have relied on overexpression of epitope-tagged proteins or fluorescent fusion proteins; additionally, most studies have used cultured cells rather than observations *in vivo*. However, there is plausible evidence that endogenous *CLN3* might reside in variable locations in different cell types. In yeast, the endogenous *CLN3* locus has been tagged by recombination which revealed that the protein appears to reside in the Golgi when expressed at endogenous levels (Kama et al., 2011); in primary neurons, overexpressed CLN3 appears to be present in synaptic vesicles in addition to lysosomes (Kyttala et al., 2004, Luiro et al., 2001); in the mouse retina one of the few studies to visualize endogenous CLN3 revealed localization to mitochondria in Müller glial cells and in photoreceptor inner segments (Katz et al., 1997). It is of interest, therefore, that we find both the endogenous *Drosophila* CLN3 and a YFP-CLN3 fusion expressed at endogenous levels are found in the apical domain of tubular structures *i.e.* exposed to the lumen, suggesting a possible role in secretion or transport. Moreover, we see also some CLN3 present in mitochondria, in agreement with expression seen in the mouse retina (Katz et al., 1997). The β-galactosidase reporter mouse of *CLN3* expression showed unexpected, strong expression from early stages of embryonic development in the endothelia of the brain (Eliason et al., 2007). It was unclear why *CLN3* is expressed there so strongly but follow-up studies from the Davidson laboratory have shown *CLN3*^*−/−*^ endothelia have disrupted membrane microdomains, altered drug efflux across the epithelium and abnormal volume regulation (Tecedor et al., 2013). Cells expressing a *CLN3* transgene after viral transduction reveal the *CLN3* protein is localized to the trans Golgi network, which regulates much of the trafficking in polarized cells. However, the cells in that study were not polarized. Could the localization of *CLN3* be different in polarised epithelia? *CLN3* is strongly expressed in renal tubules in the mouse and *CLN3* transcription is regulated by osmolality (Getty et al., 2013, Stein et al., 2010); here we show that *CLN3* is expressed in Malpighian tubules that are orthologous to the mammalian kidney. We have not asked if *CLN3* is similarly regulated by salt in fly tubules nor have we interrogated tubule function in adult flies lacking *CLN3* (or *CLN7*). However, it is potentially significant that CLN3 in *Dictyostelium* is predominantly resident in the membrane of the contractile vacuole, an osmoregulatory organelle (Huber et al., 2014). Taken together, these findings suggest a role for CLN3 in solute transport and osmoregulation. If the localization of the protein we see in the fly is conserved in polarized tubules of mammals, such as the brain endothelia, it will change our view of CLN3 protein function and the underlying cell biology of the disease.

### 4.5 CLN3 in mitochondria

Our electron microscopy study found CLN3 in multiple locations in the Malpighian tubules: in the microvilli, in mitochondria and in lysosome-like vesicles. The study that localized CLN3 to mitochondria in the mouse retina (Katz et al., 1997) has not, to our knowledge, been repeated. We are not clear whether the antibody used in that study does not recognize endogenous CLN3 in other tissues with lower expression levels - or whether it subsequently was shown to be non-specific by others. Either way, it is intriguing that we also see some mitochondrial localization of CLN3 in *Drosophila* given that an *in vitro* TAP-tag purification study identified CLN3 in complex with several mitochondrial transporters and inner membrane proteins in neuroblastoma cells (Scifo et al., 2013). How CLN3 would be imported into mitochondria is unclear. No homologue of CLN3 contains a canonical positively-charged mitochondrial import leader sequence at the N-terminus of the protein. However, only 70% of mitochondrial proteins are imported by this method and other sequences or motifs regulating import are less well described (reviewed in Chacinska et al., 2009). If a mitochondrial localization for CLN3 is confirmed *in vivo* in other mammalian tissues, it will cause us to reconsider CLN3 function.

This study highlights the power of gene editing technology in facilitating protein localization studies. We have used two different methods to study CLN3 and CLN7 that have proved difficult to study with antibodies. By removing the possibility of overexpression artefacts we have identified surprising localization for both proteins and raised new questions about the cell biology of the NCLs.

## 5 Conclusions

• Knock-in strategies in *Drosophila* were used to generate YFP-*CLN7* and YFP-*CLN3* fusions to assess *in vivo* localization without overexpression.
• YFP-*CLN7* and YFP-*CLN3* show restricted expression in the CNS. Expression is largely restricted to the surface glia that form the blood brain barrier. *CLN7* is also expressed in neurons and glia in the developing visual system.
• *CLN7* and *CLN3* are expressed in the Malpighian tubules. CLN3 is strongly enriched at the apical domain adjacent to the lumen. Some CLN3 is also detected in mitochondria.

## Author contributions

RIT and GT conceived and raised funds for the study. AM, MBO'H and RIT performed all the *Drosophila* genetics and light microscopy studies. RIT and AW performed the electron microscopy. All authors helped write the manuscript.

## Appendix A Supplementary data

Supplementary material Image 3
