# Supplementary material for: in vivo localization of the neuronal ceroid lipofuscinosis proteins, CLN3 and CLN7, at endogenous expression levels
Source: Neurobiol Dis. 2017 Jul;103:123–32. doi: 10.1016/j.nbd.2017.03.015 (PMC5441185; doi:10.1016/j.nbd.2017.03.015)
Supplement: Supplementary file 1 — Supplementary material [file mmc1.pdf]

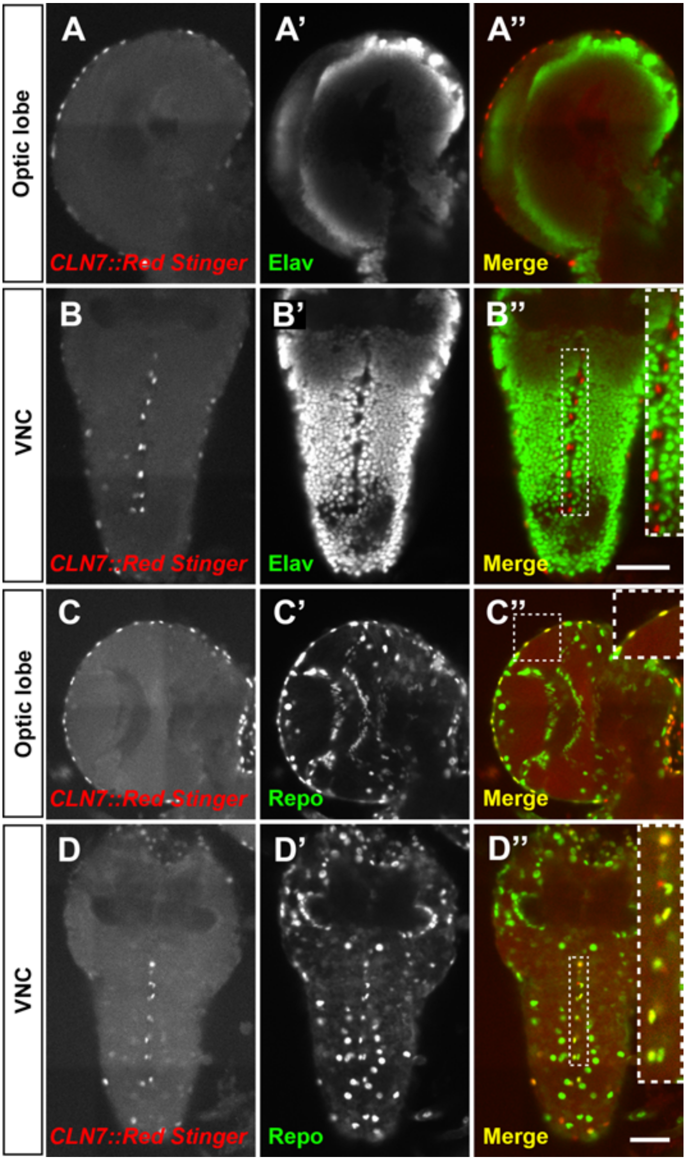


**Supplementary Figure S1**. A *CLN7* promoter reporter confirms expression in glia. CNS from *CLN7::RedStinger* flies was fixed and stained. RedStinger is a nuclear protein whose fluorescence persists after formaldehyde fixation and requires no antibody. *CLN7::RedStinger* expression (red) does no overlap with the neuronal marker anti-Elav (A,B green) but does overlap with the glial marker anti-Repo in the perineurial glia of the blood-brain-barrier and the channel glia of the ventral nerve chord (C,D, green).Scale bar = 50 μm

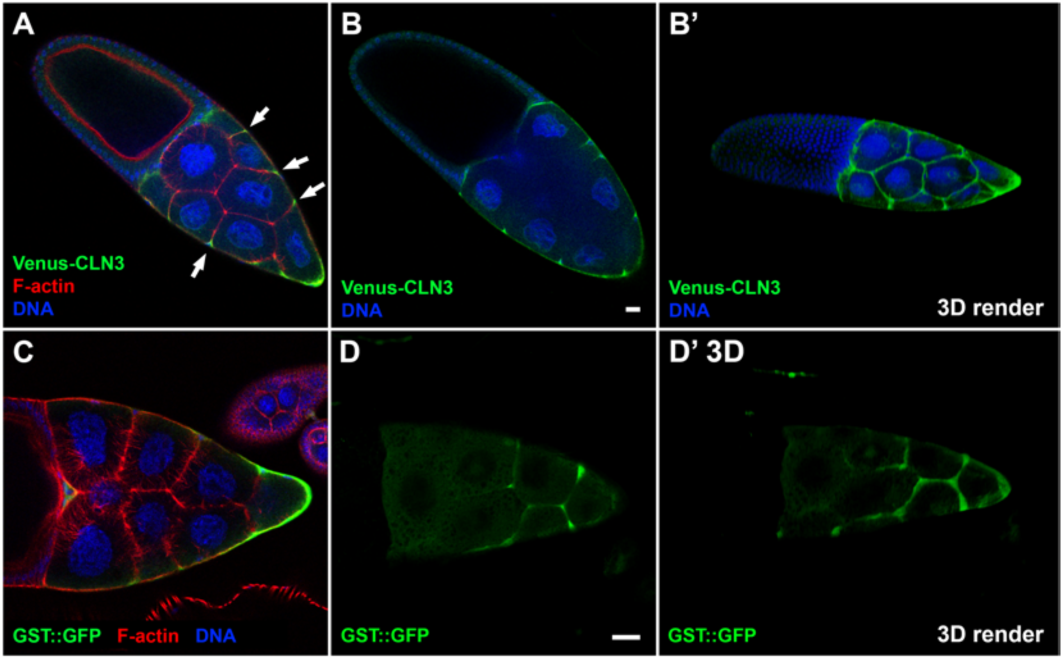


**Supplementary figure S2.** *CLN3* is expressed in the stretch follicle cells of the ovary.
A: *YFP-CLN3* expression in the stretch follicle cells that overlie the nurse cells in the developing ovary. Ovaries from fed, mated females were fixed in 4% formaldehyde in PBS mixed 1:1 with heptane for 20 mins then stained for anti-GFP (green), F-actin with Alexa 546-phalloidin (red) and DNA with ToPro3 (blue). Stretch follicle cells are shown by arrows in A. Expression begins at stage 10. B, B': single XY optical section and 3D render of a stage 10 ovary showing YFP-CLN3 and DNA only. C,D,D': YFP-CLN3 expression correlates temporally and spatially with upregulation in the stretch follicle cells of a *gstD::GFP* reporter of oxidative stress levels (Sykiotis and Bohmann, 2008. DOI: 10.1016/j.devcel.2007.12.002). Scale bar = 50 μm

**Supplementary table 1.** Details of primary antibodies used.

| $1^o$ Antibody | Species | Dilution | Supplier (clone or product) |
|---|---|---|---|
| GFP (recognises Venus-YFP) for IF | Rabbit | 1:4000 | AbCam (ab290) |
| GFP for ImmunoEM | Rabbit | 1:200 | ThermoFisher (A-11122) |
| Alexa-594 α-HRP | Goat | 1:400 | Jackson ImmunoResearch (123-585-021) |
| Elav | Rat | 1:25 | Developmental Studies Hybridoma Bank (7E8A10) |
| Repo | Mouse | 1:5 | DSHB (8D12) |
| Prospero | Mouse | 1:250 | Dr Alicia Hidalgo, DSHB (MR1A) |
| CLN3 | Rabbit | 1:500 | Tuxworth et al, 2009 |
| Discs large | Mouse | 1:25 | DSHB (4F3) |
| $Na^+K^+$ ATPase α-subunit | Mouse | 1:5 | DSHB (A5) |
